# Spatial proteomics reveals human microglial states shaped by anatomy and neuropathology

**DOI:** 10.21203/rs.3.rs-2987263/v1

**Published:** 2023-06-02

**Authors:** Dunja Mrdjen, Meelad Amouzgar, Bryan Cannon, Candace Liu, Angie Spence, Erin McCaffrey, Anusha Bharadwaj, Dmitry Tebaykin, Syed Bukhari, Felix J. Hartmann, Adam Kagel, Kausalia Vijayaragavan, John Paul Oliveria, Koya Yakabi, Geidy E. Serrano, Maria M. Corrada, Claudia H. Kawas, Christine Camacho, Marc Bosse, Robert Tibshirani, Thomas G. Beach, Michael Angelo, Thomas Montine, Sean C. Bendall

**Affiliations:** 1 Department of Pathology, Stanford University, School of Medicine, Palo Alto 94304, CA, USA; 2 Department of Neurology, University of California, Irvine, 9269, CA, USA; 3 Systems Immunology and Single-Cell Biology, German Cancer Research Center (DKFZ), 69120, Heidelberg, Germany; 4 Banner Sun Health Research Institute, Sun City, 85351, AZ, USA

## Abstract

Microglia are implicated in aging, neurodegeneration, and Alzheimer’s disease (AD). Traditional, low-plex, imaging methods fall short of capturing *in situ* cellular states and interactions in the human brain. We utilized Multiplexed Ion Beam Imaging (MIBI) and data-driven analysis to spatially map proteomic cellular states and niches in healthy human brain, identifying a spectrum of microglial profiles, called the microglial state continuum (MSC). The MSC ranged from senescent-like to active proteomic states that were skewed across large brain regions and compartmentalized locally according to their immediate microenvironment. While more active microglial states were proximal to amyloid plaques, globally, microglia significantly shifted towards a, presumably, dysfunctional low MSC in the AD hippocampus, as confirmed in an independent cohort (n=26). This provides an *in situ* single cell framework for mapping human microglial states along a continuous, shifting existence that is differentially enriched between healthy brain regions and disease, reinforcing differential microglial functions overall.

## Introduction

Microglia, the resident macrophages of the brain, are highly dynamic cells that adapt to their ever-changing environment and perform a vast array of functions spanning brain development, adulthood, aging, and neurodegenerative and neuroinflammatory diseases^1^. In aging and disease microglia can assume hypo- or hyperactive states that fail to perform their functions or become pathogenic and aberrantly engulf synapses, thus contributing to cognitive decline^2–4^. Corresponding to their diverse roles, microglia can assume a wide range of phenotypes across contexts and brain regions. Given the relative ease of targeting microglia^5^, a reprogrammable, renewable and replaceable cell type versus neurons themselves, their cellular state diversity is key to identifying their spectrum of function. Historically, microglia were defined by their morphology and later by expression of a few immune markers that defined a “bad” (M1) or a “good” (M2) state^6^. In recent years these definitions have expanded enormously. A myriad of single-cell proteomic^7–9^ and transcriptomic phenotypes of microglia have been categorized and named including in disease: DAMs^10^, MGdN^11^, GAMs^12^, ARMs and IRMs^13^, HAMs^14^, MIMs^15^, LDAMs^16^, and in development and aging: WAMs^17^, ATMs^18^ and PAMs^19^. While they have unique names, these cell state descriptions can overlap significantly, demonstrating the plasticity of microglial functions with clearly discriminating phenotypes yet to be determined. At the same time, it is accepted that microglia exhibit variability between healthy brain regions and even between layers of neurons within a region^20–26^. Particularly in the human, the binning of microglia into disparate contextual subtypes, even when captured from different brain regions under different contexts, has led to confusion in the field, and a more unifying approach to describing these cellular states is required^27^.

Computational tools typically used to separate microglial states include clustering algorithms that were developed to identify discrete cell lineages^7,28^. Much as the case for myeloid cells in the periphery, these tools fail to capture the range of overlapping microglial phenotypes presumably linked to functional identity. This is further compounded by most studies of microglia being performed in the mouse where there is growing evidence that their diversity does not overlap with that of humans^29^. Existing human brain samples have been limited in the anatomical regions analyzed, cell number acquired, or methods targeting nuclear rather than cytoplasmic transcripts^30–32^. Analysis of single-cell suspensions leaves spatial information behind as they require extraction of cells instead of *in situ* analysis within the microenvironment which further informs function. Spatial transcriptomics^33^ and immunohistochemistry studies^34,35^ of microglia to date have been restricted by relatively little human data, low resolution, low multiplexing capabilities, and very few cells with the purpose to validate specific genes or proteins of interest found through scRNA-seq or CyTOF^12,36^, and have largely lacked quantitative metrics of markers and multi-cellular niche analysis.

Here we investigate human microglial phenotypic diversity at a quantitative, spatial proteomic, and morphological level within different regions of the human brain in post-mortem formalin-fixed paraffin embedded (FFPE) tissue from individuals enrolled in a research study who were repeatedly and carefully annotated by neuropsychological and clinical assessments, and ultimately by comprehensive neuropathologic evaluation. We made use of a single-cell, spatial proteomic imaging tool called multiplexed ion beam imaging (MIBI)^37,38^ to capture microglia within intricate multi-cellular microenvironments and pathologic niches of the brain. MIBI has been used previously to study various archival human tissue types from tumors^39–41^ to placenta^42^ and granulomas^43^ in infectious disease and, recently, the brain^44^. With this we created a 41-plex imaging analysis to capture major brain resident cell types, and importantly, simultaneously quantify the expression of 17 microglial immunophenotyping proteins. From our initial imaging study we extracted 203,717 myeloid cells – 199,204 were microglia, 430 were monocytes and 4,080 were vessel-associated macrophages (BAMs^7^) – across 5 brain regions from individuals with healthy brains. Instead of organizing cells into clusters, we recognized a continuous progression of cell phenotypes that we defined here as the microglial state continuum (MSC). The distribution of microglia on the MSC was anatomically restricted between healthy brain regions, and conversely, compartmentalized within local microenvironments. Contrasting the healthy brain profiles to Alzheimer’s disease (AD) hippocampus, we identified sparse DAM phenotypes enriched around amyloid plaques, and more significantly, revealed that all hippocampal microglia were polarized towards a less active MSC compared to control tissue. This shift in the AD related MSC was validated using an independent analysis of hippocampal tissue from individuals exhibiting a spectrum normal cognition and AD-related cognitive decline. Using our high parameter data as a guide, we used the combinatorial expression of HLA-DR and Iba1 to recapitulate the high dimensional MSC on an additional ~60,000 microglia sampled from 26 individuals. Here, we showed a significant shift in AD as well as an overall correlation of MSC progression with cognitive decline. Taken together, this study is the first highly multiplexed, quantitative, spatial proteomic analysis of microglial phenotypes in the human brain, creating a new, evidenced-based definition of a continuum of cell states that varies between brain regions and is skewed towards an impaired state in neurodegenerative disease.

## Results

### Multiplexed spatial proteomics organizes macro- and microenvironments in the human brain.

**Figure 1.**
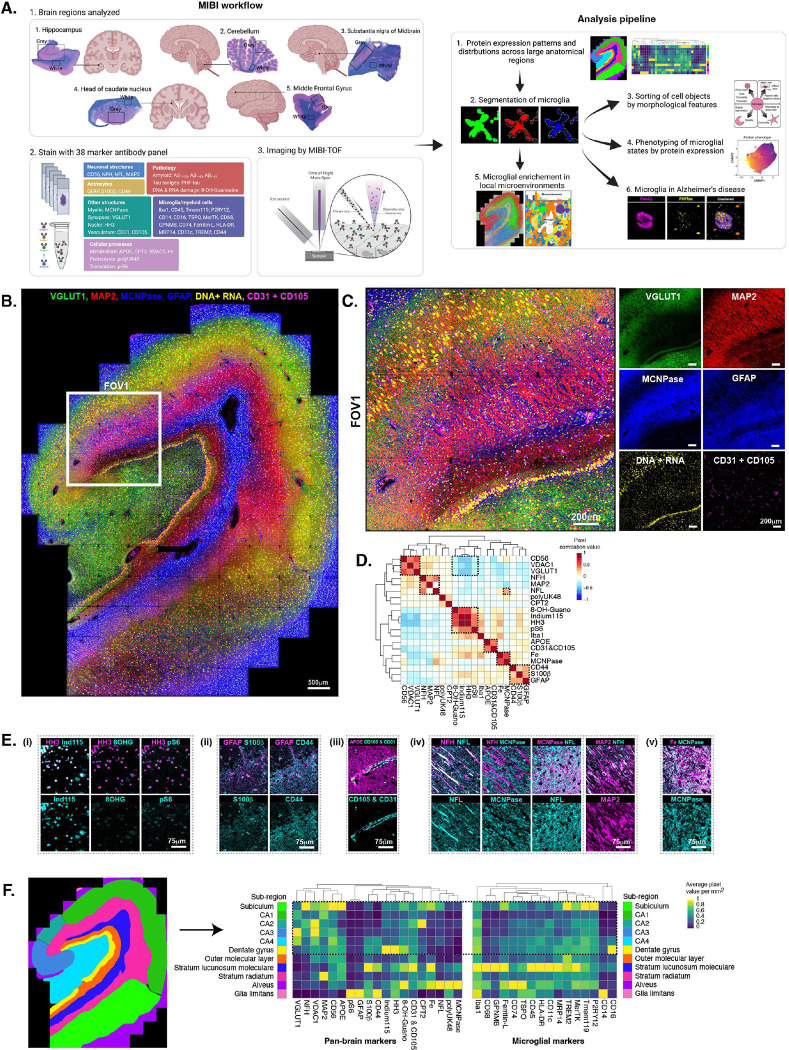
Multiplexed ion beam imaging (MIBI) maps the macro- and microenvironment of the human brain through spatial proteomics. (A) The MIBI workflow including human brain anatomical region and field of view (FOV) selection, staining with a 38-marker antibody panel and the computational pipeline spanning single-cell and niche analysis. (B) The human hippocampus imaged with 106 stitched FOVs (700 μm × 700 μm each) showing VGLUT1 (green), MAP2 (red), MCNpase and GFAP (blue), DNA + RNA (yellow, through free Indium 1153+ staining) and CD31 + CD105 (magenta). (C) An enlarged view of FOV1, the CA2 and partial dentate gyrus, shown in (B), highlighting fine cellular features including nuclei, pyramidal neuronal soma and dendrites, synaptic densities, myelin, astrocytes and vasculature. (D) Pixel correlation of pan-brain proteins used to identify multi-cellular niches in the brain, highlighting strong positively and negatively correlated protein programs. (E) Images of proteins depicted in (D) demonstrating pixel signal overlap and exclusion for (i) nuclear proteins, (ii) astrocytes proteins, (iii) vascular proteins, (iv) axonal and dendrite proteins in neurons, and (v) endogenous iron (Fe). (F) Sub-regional organization of the human hippocampus from expert neuropathological annotation (left) and the protein abundance in each sub-region (right) calculated as average pixel value per mm^2^, with the inner cornu ammonis, subiculum and dentate gyrus areas highlighted.

Most human brain samples exist in archival FFPE blocks. Immunohistochemistry (IHC) of archival FFPE tissue is challenging when multiplexing is introduced. Fluorescent IHC, particularly the brain, is confounded by high background autofluorescence that becomes even more problematic with multiplexed probes. In order to reliably study complex microglial states *in situ* across the human brain, we implemented MIBI technology that uses metal labelled probes and does not have the same constraints as fluorescent or dichromatic IHC. We started by targeting five brain regions from one healthy aged donor (90-year-old male without dementia, *APOE* ε3/ε3, and a post-mortem interval of 3.4 hours), including the hippocampus (HIP), cerebellum, substantia nigra (SN), caudate nucleus, and middle frontal gyrus (MFG) (Figure 1A, **left**). Sections were stained with a panel of 38 metal-tagged antibodies (**Extended Data Table S1**) capturing neuronal structures including soma, dendrites, axons, synapses, astrocytes, myelin, vasculature, cellular processes including metabolism, proteolysis and translation, the hallmark AD neuropathologic changes (ADNC) including amyloid-β (Aβ) plaques and tau tangles, and a sub panel of 17 microglial phenotyping proteins. Additionally, we stained nucleic acids (DNA & RNA) using free indium 115^3+^ ions^45^ and captured endogenous iron (Fe), an important marker of brain health^46^ and microglial state^47^. Areas outlined in black within each Hematoxylin-Eosin (H&E) and Luxol Fast Blue (LFB) stained section were imaged by MIBI by tiling multiple images of 700 μm × 700 μm each, including larges area of mostly grey matter (labelled “Grey”) as well as smaller areas within the deep white matter (labelled “White”). The data analysis was a multi-pronged approach including quantification of protein expression patterns in large demarcated sub-regions, segmentation of microglial cells, *in silico* sorting of morphological subtypes, phenotyping of microglial single cells and evaluating changes across large brain regions, pixel clustering of local microenvironments and spatial analysis of niche enrichment of microglial states, and comparison to an AD dementia case (Figure 1A, **right**).

Approximately 6.5 mm × 5.5 mm of the hippocampus was imaged at ~470 nm pixel resolution (Figure 1B). Exemplary stitched overlays of the hippocampus show markers VGLUT1 (excitatory synaptic density), MAP2 (neuronal dendrites and soma), MCNPase (myelin), GFAP (astrocytes), DNA & RNA (nuclei and neuronal soma as stained by free indium 115^3+^ metal ions) and CD31 & CD105 (vasculature). The boxed FOV1 in larger format overlay (Figure 1C) of the same image highlights the large areas of synaptic density, myelin, astrocytes as well as the fine structures of dendrites, soma of the hippocampal pyramidal neurons and granule cells of the dentate gyrus (DG), nuclei and vasculature. Next, in order to assess the biological validity of pan-brain proteomic targets, separate from those used for microglial phenotyping, we correlated expression at the pixel level within the entire hippocampus (Figure 1D). As anticipated, nuclear markers correlated highest with other nuclear markers (HH3, Indium115^3+^, 8-OH-Guanosine (a marker of DNA & RNA damage), p-S6 (a surrogate marker for mTOR pathway activity), see image panel (**D i**)), astrocyte markers correlated positively with other astrocyte markers (GFAP, S100β, CD44, (**D ii**)) although not highly since these proteins are expressed in different sub-cellular locations of astrocytes, and VGLUT1 correlated well with other widely dispersed “carpet” markers like CD56 (aka NCAM1, expressed by neurons and glia) and VDAC1 (a mitochondrial protein) and anti-correlated with nuclear markers. ApoE is produced primarily by astrocytes and microglia and by some neurons in response to injury, and contributes to integrity of the blood brain barrier (BBB)^48^. In our images, ApoE was widely dispersed and concentrated at blood vessels (**iii**). Axonal proteins, neurofilament (NF) high (H) and low (L) molecular mass, correlated positively with each other, NFH with MAP2 as well and NFL with MCNpase (**iv**), although only in specific sub-regions of the hippocampus while in other sub-regions the proteins were not co-expressed. Fe correlated highly with the myelin enzyme MCNPase, also in specific sub-regions (**v**). PolyUK48, a protein upregulated during the proteolysis, correlated positively with CPT2 (carnitine palmitoyltransferase 2) and Iba1 corelated positively with Fe and nuclear markers. Overall, our pan-brain markers correlated with biologically expected sets of proteins and built confidence that our method for interpreting the brain through MIBI is sound and not confounded by technical artifacts.

To further analyze the regional distribution of these markers in the hippocampus, given its familiar differential protein expression across its sub anatomy, we demarcated the sub-regions based on well documented neuropathological annotations including the subiculum, cornu ammonis (CA1–4), DG, outer molecular layer, stratum lacunosum moleculare, stratum radiatum, alveus and glia limitans (Figure 1F). The density of expression of brain and microglial immunophenotyping proteins in these local sub-regions was quantified as average pixel expression per mm^2^, with the inner regions of the hippocampus highlighted. The subiculum and CA1–4 were enriched for neuronal and synaptic markers like VGLUT1, NFH, MAP2 and CD56, VDAC1 and ApoE. Interestingly, CA2, a subregion of hippocampus relatively resistant to AD-type changes, had a high level of CPT2. The DG is compacted with granule cell neurons, as evidenced by the high expression of HH3, 8-OH-Guanosine and the binding of indium115^3+^. Moving from the DG through the outer molecular layer, there is a distinct diminishing of nuclear markers and pan-brain marker expression. The stratum lacunosum moleculare was defined by a high density of astrocyte soma and blood vessels as seen by the strong expression of S100β and vascular markers CD31 & CD105. While the stratum radiatum that harbors radiating dendrites from the CA1 area displayed the highest expression of MAP2. The alveus which is compacted with axons is highlighted by dense MCNpase protein. Finally, the glia limitants, the astrocytic barrier covering the brain, is enriched in GFAP and CD44 as well as pS6 expression. Interestingly, microglial phenotyping marker density was lower in CA1 compared to neuronal and synaptic markers. Differential expression of microglial markers was observed between subiculum (P2RY12, Tmem119, TREM2), stratum lacunosum moleculare (Iba1, CD68, GPNMB, Ferritin-L, CD45, HLA-DR, CD11c, MRP14, MerTK) and alveus (CD74, TSPO). The DG and glia limitans had the highest expression of CD16 and CD14, respectively.

Beyond our ability to capture expected neurobiology and co-localization, this multiplexed, quantitative spatial proteomic analysis within hippocampal subregions uniquely captures compartmentalization of different neuronal, structural, and glial features including differential densities of microglial phenotyping proteins across this important brain region. Further to this, spatial proteomic analysis in the MFG, cerebellum, SN, and caudate (**Figure S1A-D**) was able to obtain similar correlations of subregional compartmentalization to quantified features.

### Quantifying proteomic expression profiles on microglia with spatial-anatomical context.

**Figure 2.**
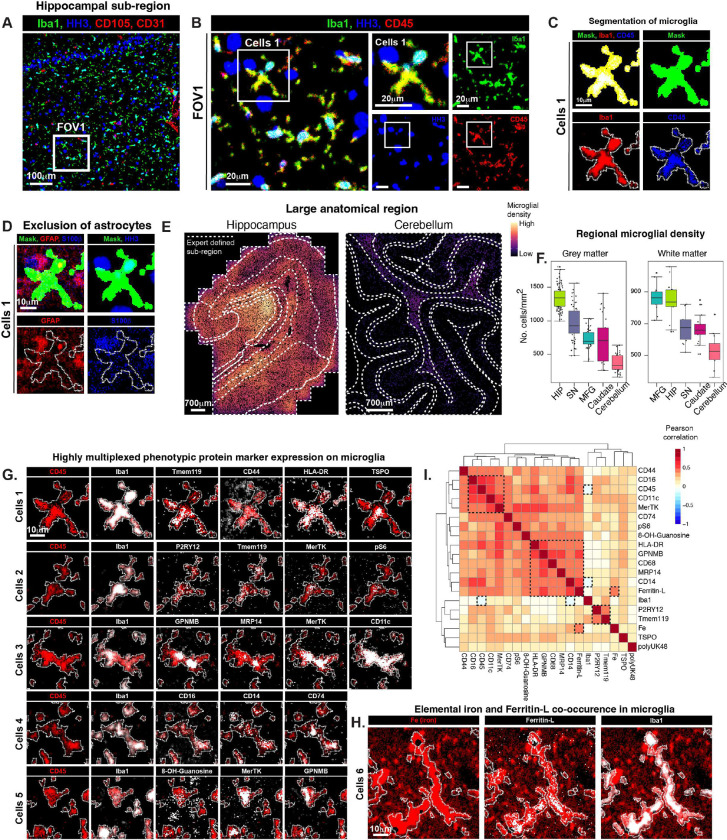
Microglial segmentation and phenotyping within the context of large anatomical brain regions. (A) Iba1^+^ (green) microglia depicted in a single FOV from the human hippocampus in a < 1 mm depth of field, with nuclear staining by histone H3 (HH3, blue) and vascular staining by CD31 + CD105 (red). (B) An enlarged view of FOV1 in (A) highlighting a microglia cell (Cells 1) positive for Iba1 (green) and CD45 (red) with its HH3^+^ (blue) nucleus in the plane. (C) Microglia were segmented with eZsegmenter by creating a hybrid mask (green) based on Iba1 (red) and CD45 (blue) expression, normalized for cell size. (D) Inclusion of microglial nuclei (HH3, blue) from the segmentation mask and exclusion of other glial markers like GFAP (red) and S100β (blue). (E) Microglial cellular density in local expert-annotated sub-regions across the hippocampus and cerebellum, depicted as color overlay on dots (microglia) across each stitched image. (F) Quantification of local microglial density in each acquired FOV for each large anatomical brain regions in grey and deep white matter areas, including the hippocampus (HIP), substantia nigra (SN), middle frontal gyrus (MFG), caudate and cerebellum (n = 1 for each brain region). Box and whisker plots represent the minimum, first quantile, median, third quantile and maximum, with individual points representing FOVs. (G) Microglial phenotyping protein expression within segmented cellular masks across five sets of single cells showing differential intracellular localization of each protein. (H) Endogenous elemental iron (Fe) found within and outside of segmented microglia and its colocalization with Ferritin-L. (I) Pearson correlation of microglial phenotyping proteins at a cellular level, with specific positively and negatively correlated protein programs highlighted.

With the faithful quantification of multiplexed protein expression in different neuroanatomical spaces and subregions at a pixel and bulk level, we sought to identify discrete microglial cells and define their distribution within the different subregions. To this end, we used classical microglial markers Iba1 and CD45 and, leveraging the high resolution of our images, we were able to capture microglial cells and parts of cells. Figure 2A shows a single 700 μm × 700 μm image from the hippocampus with larger format in Figure 2B highlighting a single microglial cell.

In order to phenotype microglia at a single cell level, we used an intensity thresholding-based segmentation approach as a part of the MIBI image processing toolkit (see methods). Microglia were defined as Iba1^+^ and/or CD45^+^ and a composite channel mask was created with which a total of 203,714 microglia (with BAMs and monocytes) were extracted from our images and pre-processed for single cell analysis (Figure 2C, **S2A**). Microglial cell extraction largely excluded pixels from other glia, for example astrocytes (Figure 2D), although these pixels were present to some extent as cells appear to overlap in the collapsed *Z* plane. Microglial cell density across all the large brain regions was quantified as the number of cells per mm^2^ per FOV, within grey and deep white matters (Figure 2E, F, **S2B**) which showed the highest density of microglia in the hippocampus, particularly in the grey matter (here defined as the entire area shown in Figure 2E, including the white matter tracts, and separate from the deep white matter areas imaged separately); the cerebellum had the lowest microglial density and the SN, MFG and Caudate showed intermediate expression. Striking differences in microglial density between the hippocampus and cerebellum is better appreciated in the overlaid images shown in Figure 2E, where each dot represents a microglial cell colored by its local density in a 4.9 μm × 4.9 μm area. Notably, microglial density was particularly compartmentalized to sub-regions within the hippocampus and cerebellum, both allocortical areas (Figure 2E, **S2B**). For instance, the microglial density in the cerebellum was highest in the local myelin layer, corresponding to the high pixel density of glial markers in our annotated sub-region analysis of the cerebellum (**Figure S1B**), and lowest in the molecular layer and granular where there are relatively sparse VGLUT1^pos^ synapses and dense HH3^pos^ CD56^pos^ neuronal soma (**Figure S1B**). In the hippocampus, microglial density was highest in CA4 and CA2, where MAP2^pos^ axons are sparse and synapses are very dense, and lowest in CA1, outer molecular layer, and subiculum where MAP2^pos^ axons are dense, and synapses are sparser. Microglial density was also relatively high in the hippocampal myelin and astrocyte rich areas, as in the cerebellum. These patterns of microglial density being enriched in areas of dense synapses and dense myelin are in accordance with their roles in synaptic pruning^49^ and maintaining myelin integrity^50,51^ in concert with astrocytes.

Interestingly, expression of our microglial phenotyping proteins within our segmented cells varied and was localized to different intracellular niches within microglia (Figure 2G). For instance, where Iba1 and CD45 were observed within the entire cytoplasm of the cell, Tmem119 (considered a bona fide microglial marker in mice^52^) was only present in some areas of the cell, leaving whole segments unmarked. P2RY12, also an exclusively microglial marker in the brain, had a more homogenous intracellular distribution but its expression was generally low. HLA-DR and CD11c were highly expressed throughout the cell, while MRP14 and GPNMB tended to be found in bright, dense intracellular spots, as were MerTK and CD74. CD44 was expressed within microglia and outside of microglia in astrocytes. The product of free radical damage to nucleic acid, 8-OH-Guanosine, was also not restricted to microglia. Endogenous Fe was present in large areas that we imaged, particularly in the hippocampus and SN. Amongst other cell types, Fe was localized to microglia and often co-localized with Ferritin light chain (Ferritin-L, Figure 2H). Correlation of these features across all microglia extracted from all brain regions on a cell-level revealed programs of co-expression (Figure 2I). Iba1, however, was not highly correlated with any other marker in the panel; in fact, it was negatively correlated with CD45 and CD14 and only slightly positively correlated with Tmem119. The two “homeostatic” markers for microglia, P2RY12 and Tmem119, correlated most highly with each other. Fe had a moderately high correlation with Ferritin-L, consistent with it being a marker of iron storage in microglia^53^. Amongst the positively correlated set of markers, CD16 (also known as FcγRIII), CD45, CD11c and MerTK formed a subset, and HLA-DR, GPNMB, CD68, MRP14 (all implicated in phagocytosis), CD14 and Ferritin-L formed another subset. MerTK, also a protein involved in phagocytosis, was promiscuously correlated with both subsets. TSPO, a marker routinely used in PET imaging to evaluate microglial activation in people and animal models which has been shown rather to reflect microglial density^54,55^, was not highly correlated with any other proteins in this panel; and polyUK48 even less so. Taken together, MIBI imaging of microglia across the human brain allows for robust, multiplexed, single cell phenotyping and local cellular density analysis where the quantitative nature of the data allows us to reveal disparities in microglial diversity not previously realized.

### Classification of microglia based on multiplexed morphological features separates nucleated cells and fragments, but not anatomical location

**Figure 3.**
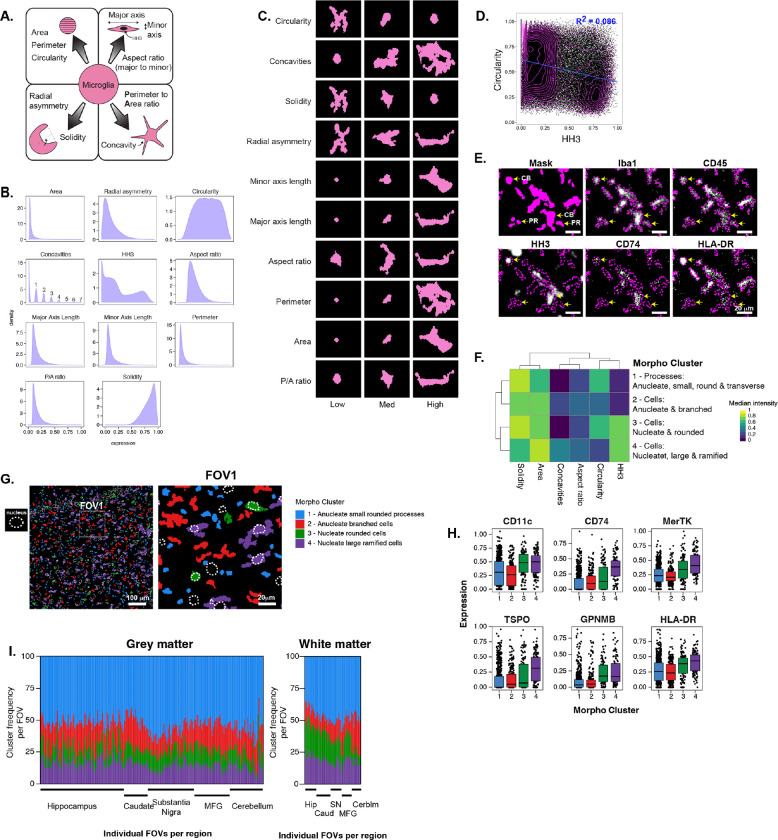
Classification of microglial cells and cell fragments using multiplexed morphological features. (A) Multiplexed morphological features extracted with eZsegmenter including metrics of cell size. (B) Aggregated data spread of microglial morphological features across all brain regions, including both grey and deep white matter. P/A ratio: perimeter to area ratio. Each dot represent a FOV taken from one brain region. (C) Single-cell representative images on microglial masks of each morphological metric spanning low, middle and high levels. (D) The correlation of nuclear signal as measured by single-cell HH3 expression and circularity across the entire microglial data set is weak and reveals heterogeneous populations of differential nuclear inclusion and circularity levels. (E) Representative images of differential protein localization within circular cell bodies (CB), larger cytoplasmic cell pieces and small round processes (PR) within the segmented microglial mask (magenta fill and dashed magenta line). (F) Four FlowSOM morpho clusters calculated from the entire microglial data set using select morphological features uncovers nucleate and anucleate cells and cell parts with varying degrees of branching and size. (G) Clustered overlays of the four morpho clusters within a single hippocampal FOV, highlighting an enlarged area (FOV1) and the presence or absence of a nucleus in the plane (dashed white line). (H). Differential protein expression of select microglial phenotyping proteins in each of four morpho clusters. (I) Distribution of the four morpho clusters in each individual FOV acquired for each large anatomical brain region across grey and deep white matter. MFG: middle frontal gyrus.

In the absence of distinct markers for microglial states, morphology has been historically used in an attempt to classify these cells as resting/homeostatic (ramified with long, fine processes), activated (rounded, amoeboid, large, thickened), dystrophic (fragmented, distorted) and many states in between^56,57^. Now that we were able to segment microglia and assess their intracellular and intercellular protein expression, we set out to define their morphological states and determine whether this morphological classification could inform protein expression, or vice versa. We extracted a set of morphological features from our 2D segmented microglia (Figure 3A), including size (area, perimeter), circularity, elongation (major and minor axis length, aspect ratio), cell border undulation (perimeter to area ratio (P/A ratio); number concavities), and symmetry (radial asymmetry, solidity), as well as the presence of a nucleus in the plane of view. Many morphological features were highly correlated with each other (positively and negatively) (**Figure S3A**), shown in representative cells at low, medium and high values for each feature (Figure 3C). Cells tended to be similar in size, many had zero concavities, others ranged from one to seven concavities; circularity was also widely distributed; HH3 nuclear signal had three major peaks likely corresponding to how much nucleus was in the plane (Figure 3B).

Cell objects that did not have any HH3 signal and were thus anucleate, were likely pieces of cytoplasm. In order to separate very small cell objects that were likely parts of microglial processes from larger cellular areas, we postulated that transversely sectioned processes would be small, round and anucleate. Indeed, size and circularity were negatively correlated. However, circularity was not highly correlated with HH3 and there were four distinct populations of HH3 signal and circularity (Figure 3D), suggesting that we captured parts of processes (PR), irregularly shaped anucleate cell objects, rounded nucleate cells with their cell body (CB) in the plane, and irregularly shaped nucleate cells (Figure 3E). All cell objects had Iba1 and/or CD45 expression throughout their cytoplasm but some markers, like CD74 and HLA-DR, were very low in small round processes. We clustered our microglia by morphological features using FlowSOM^58^ and performed manual meta-clustering into 4 main morpho clusters (Figure 3F, G): anucleate, small, rounded and transverse processes high in circularity and solidity; (2) anucleate, branched cells that were larger and had some concavity; (3) nucleate, rounded cells; and (4) nucleate, large, even more branched cells. The four ‘morpho’ clusters differed in protein marker expression for a few of our phenotyping proteins, where particularly the anucleate small, rounded processes and, to a lesser extent the anucleate branched cells, had lower expression of CD11c, CD74, MerTK, TSPO and GPNMB, and HLA-DR (Figure 3H, **S3B**). Since we had seen that an overlapping set of proteins with these tended to be found in very localized intracellular niches (Figure 2C), when the cell body of a given microglia was largely beyond our rastered plane we therefore would not be able to capture that protein reliably.

We also calculated frequency of morpho clusters per FOV acquired in each brain region (grey and white matter) but found no significant enrichment across brain regions (Figure 3I). This seems to indicate that healthy brain tissue is relatively uniform in its microglial morphological composition, and that the variance we see here is a combination of normal biology with the technical limitation of our shallow (< 1 μm) imaging plane. Furthermore, our analysis of microglial morphological features allowed us to *in silico* sort cells and cell parts to select reliable morpho clusters for protein detection. Therefore, we excluded morpho cluster 1 – small, rounded, transverse processes – from subsequent cellular phenotyping.

### Microglia align along a continuum of phenotypic cell states which are enriched in specific brain regions.

Phenotyping microglia through clustering methods has been employed across disease contexts in transcriptomic and proteomic data, resulting in the identification of sometimes distinct populations in murine models^7,10^ but often, especially in human studies, seemingly arbitrary boundaries are drawn between clusters that are very similar to each other in the high dimensional space^9,12,31^. We sought to organize these microglial states based on protein expression across brain regions without forcing cells into static, somewhat arbitrary, clusters. Excluding small cell pieces by excluding morpho cluster 1 (**Figure S4A**) left 93,679 cells. We removed sparse monocytes found in blood vessels that would otherwise contaminate our analysis. We identified intravascular monocytes as MRP14^high^, round, nucleate cells found close to CD31^+^ & CD105^+^ blood vessels (**Figure S4B**) which we gated out, leaving a total of 93,325 microglia from all tissues for analysis.

In our initial attempt to organize these cells, we used FlowSOM to identify 12 clusters of microglia. However, while different in magnitude, these clusters had similar protein expression profiles overall, resembling a continuum of states, where cluster 1 had overall low expression and cluster 12 had overall high expression across many proteins associated with microglial function (**Figure S4C**). Most cells landed in the mid-expression clusters 5, 2 and 10 (**Figure S4D**). Further, dimensionality reduction showed a progression of clusters from 1 to 12 in embedded UMAP space, again resembling a continuum of states rather than distinct populations (**Figure S4E**). As the boarders of cell clusters seemed inseparable in high dimensional space, there was concern it would create hazard in mapping meaningful changes between clusters and brain regions or disease.

**Figure 4.**
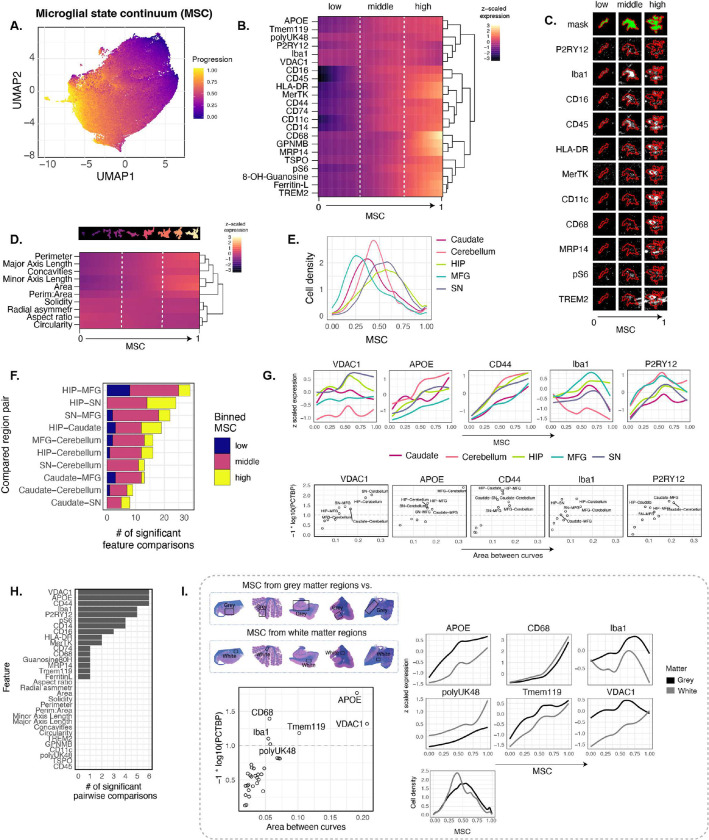
High-dimensional single-cell proteomic phenotyping of microglia through trajectory analysis across healthy human brain regions. (A) Dimensionality reduction of all extracted microglia from all brain regions (from one healthy donor) with UMAP and progression along the microglial cell state continuum (MSC) calculated by SCORPIUS pseudotime using only protein features. (B) Differential relative protein expression for each phenotypic protein along the MSC from low, medium and high MSC. (C) Representative images of single cells and their expression of protein markers from low, medium to high MSC within the segmented mask (green fill, red dashed line). (D) Progression of relative expression of morphological features across the MSC calculated with protein features with representative images of cells. (E) Distribution of microglial cellular density in each large brain region across the MSC from one healthy donor. (F) Total number of statistically significant differences between feature comparisons (including both protein and morphology) between each brain region pairwise comparison in binned low, middle and high MSC. (G) Expression of the five most highly statistically significant differentially expressed features between brain regions along the MSC (top), with their statistical significance represented as their Pairwise Comparison of Trajectories by Binned Permutations (PCTBP) score (significant above a threshold of 1) and area between the curves for each brain region pairwise comparisons (bottom, shown as a single open circle for each pair). (H) Summary of the individual features (protein and morphology) and their total number of significant pairwise comparisons for each brain region pair. (I) Features that are significantly differentially expressed along the MSC in grey (black line) vs. deep white (grey line) matter areas depicted in volcano plot with individual proteins and their relative expression along the MSC, as well as the microglial cellular density of grey and deep white matter along the MSC.

As such, we sought to embrace the continuous nature of these microglial states for quantification and comparison rather than binning into distinct populations. To this end, we employed SCORPIUS^59^, a computational method for trajectory modeling to generate a pseudotime calculated using protein features only which aligned our cells along a trajectory we termed the microglial state continuum (MSC) (Figure 4A). The MSC followed the original cluster progression along the UMAP embedding. Since the hippocampus region had the largest imaged area, and therefore greatest number of cells (**Figure S4F**), we wanted to confirm that the MSC was not skewed by this region and that cell states from less abundant regions were still captured. Therefore, we compared single, separately calculated, brain-region MSCs to that calculated form combined regions and found that they had strong agreement with each other (**Figure S4G**). To further assess whether subsets of the MSC calculated with all cells is biased towards a specific brain region, we generated a simple linear regression model of the MSC using a 70–30 train-test split and compared the test error (squared residuals) of cells in each region along the MSC. There was no region-specific difference in error for any region along the MSC, and the smoothed error lines for each region were overlapping (**Figure S4H**). From this we concluded that our approach of calculating an MSC trajectory as a quantitative summary of cell identity was a robust approach to capture and compare high dimensional microglial phenotypes from both abundant and sparsely distributed tissues.

Interrogating protein expression, which was normalized to extracted cell size (Figure 4B, **S4J**), along the MSC showed that expression of many molecules increased from low to high levels as the MSC progressed, particularly HLA-DR, MerTK, CD11c, MRP14, TREM2, etc. Other proteins like P2RY12 showed parabolic expression, high in the middle and low in the ends of the MSC. Iba1, on the other hand, fluctuated more than other markers, decreasing with increasing MSC. Representative overlay of marker expression is shown for individual cells within low, middle and high MSC (Figure 4C). Overall, low MSC cells appeared to be expressing less protein and late MSC cells appeared to be expressing more protein overall, suggesting that low MSC represents a quiescent, potentially senescent, cell state while middle to high MSC represents more active, but not necessarily activated, cellular states.

We also assessed MSC calculation with morphological features (MSC-morph). The MSC-morph progression (**Figure S4I-J**) failed to organize proteomic features and resulted in high variation making the correlation to the clear proteomic spectrum low (**Figure S4K**). This discrepancy shows that morphology alone do not organize microglia into a unified spectrum of cell states. One caveat to this is the shallow depth of our imaging plane (< 1 μm), thus we may not have sufficient cell area alone to capture their morphology robustly. While the MSC we moved forward with was calculated using only the protein features of cells, morphological features were somewhat enriched where the low MSC cells were on average small with fewer concavities and potentially dystrophic, and late MSC cells were larger with more concavities and potentially more active cells (Figure 4D, **S4J**), aligning with our interpretation of the proteomic expression features overall.

Next, we sought to reveal whether microglia differed in their cell states along the MSC between different brain regions. Cell density along the MSC was generally highest towards the middle of the trajectory and lowest at the extremes (Figure 4E). Microglia from the MFG and caudate were skewed towards the low MSC, suggesting less activation for microglia in those brain regions. Cerebellar microglia had the highest enrichment in the middle compared to other brain regions, with fewer highly activated or highly inactivate microglia. The hippocampus and SN were skewed towards the high MSC, suggesting that microglia in those regions are on average more active. Given these brain region-specific differences in cell distributions, we next asked whether there were significant differences between the protein markers along the MSC of cells from different brain regions. To this end, we designed a non-parametric method for calculating differences between trajectories from single-cell data which we termed Pairwise Comparison of Trajectories by Binned Permutations (PCTBP, **Figure S4J, *see*** methods). To show these differences, we summed the number of significant feature comparisons for each brain region pair in equally divided low, middle, and high MSC phases (Figure 4F). The hippocampus and MFG had the most differences between features while the caudate and SN had the fewest. Feature differences between brain regions were concentrated in the middle MSC with fewer differences in the low and high MSC suggesting that the phenotypic state of intermediate activation of microglia was most highly variable among brain regions. Interestingly, even though the hippocampus and SN had a similar distribution of cell density along the MSC, they still had the second highest number of phenotypic feature differences, with a large fraction of those being in high MSC, suggesting that while hippocampal and SN microglia may be skewed towards a more active state, this state is not necessarily of the same high dimensional phenotype. The top five variable features between brain regions were protein markers (Figure 4H), namely VDAC1, APOE, CD44, Iba1 and P2RY12, and are shown individually as z-scaled progressions along the MSC (Figure 4G, *top*) as well as their PCTBP significance scores for each brain region pair (Figure 4G, *bottom*), and all unscaled inter-regional levels of protein and morphological features along the MSC in order of statistical significance (**Figure S4J**). Overall, protein features were more varied between brain regions while morphological features were very similar for all brain region pairs across the MSC (Figure 4H). Since microglia from white and grey matter perform different functions and have been shown to assume different states^36^, we also compared the MSC from areas of exclusively white matter vs. grey matter that were in the same tissue blocks as the regions of interest and found that, again, only protein features varied between regions, including APOE, CD68, Iba1, polyUK48, Tmem119 and VDAC1 (Figure 4I, **S4K**). Grey matter microglia had higher APOE, Tmem119 and VDAC1 expression while white matter microglia had higher CD68 and poyUK48 expression, proteins linked to phagocytosis and protein degradation, respectively.

Overall, phenotypic differences of microglia between brain regions and white or grey matter along the MSC were subtle but significant. Our data allowed us to not only detect marker expression but quantify relative expression between cells and thus revealed changes across many proteins that may have gone unnoticed by more binary methods. The differences between low and high MSC microglia may not be picked up by standard clustering methods since all cellular states between low and high MSC are also present. We observe that a continuum approach to assessing microglial states is key to understanding microglial heterogeneity within the human brain where a spectrum of microglial states exists together.

### Microglial states are compartmentalized within local microenvironments of larger brain regions.

**Figure 5.**
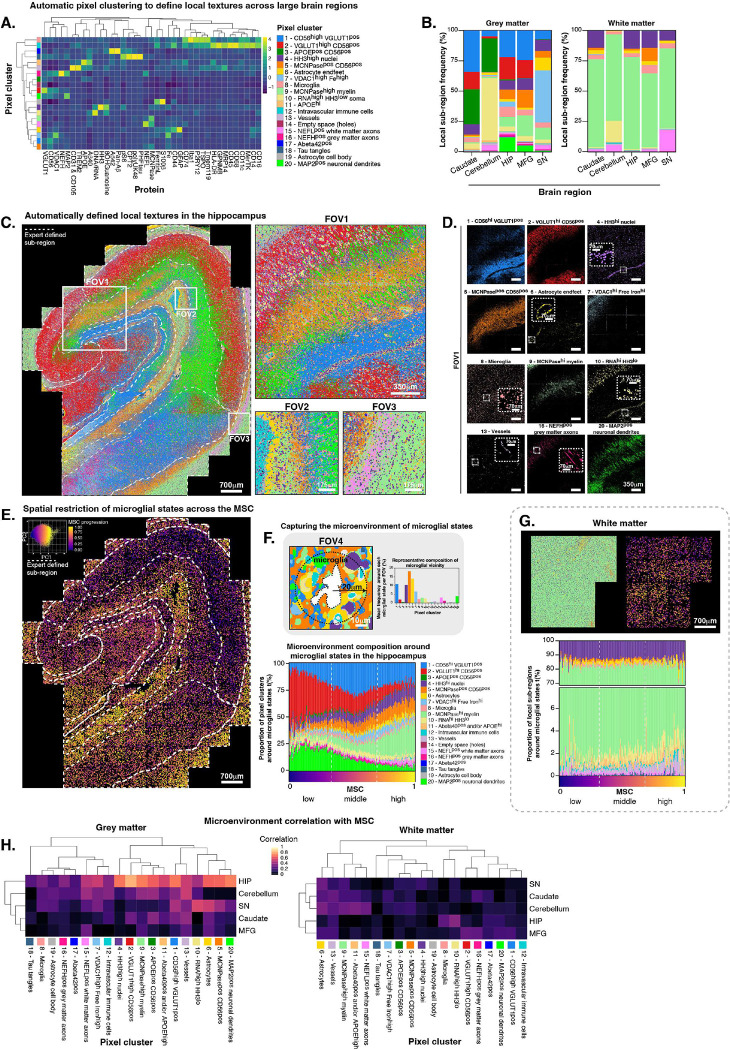
Microglial states are enriched in local microenvironments as defined by pixel clustering and neighborhood analysis. (A) Local brain textures identified through pixel clustering of all FOVs from all healthy human brain regions from a healthy donor using pan-brain protein markers, with expression of all panel protein makers for each of twenty pixel clusters. (B) Distribution of pixel clusters for each large brain region, quantified as average frequency per FOV for grey and deep white matter. (C) Spatial compartmentalization of overlaid pixel clustered brain textures in the hippocampal local sub-regions with enlarged FOVs (right): FOV1 depicting grey matter with high synaptic density (pixel clusters 1 and 2), nuclei (pixel cluster 4), pyramidal neurons (pixel cluster 10), dendrites (pixel cluster 20), areas of grey and white matter mixtures (pixel cluster 5); FOV2: astrocyte endfeet alone vasculature (pixel cluster 6), intravascular immune cells (pixel cluster 12); FOV3: white matter axons (pixel cluster 15) and myelin (pixel cluster 9). (D) Brain texture pixel clusters present in FOV1 shown as single layer images with enlarged individual features for clarity. (E) Single microglia depicted spatially within the hippocampus and colored by their MSC value, with expert annotated subregions (dashed white line). (F) Quantification of the proximal niche around each microglia calculated as the pixel cluster frequency within a 20 mm radius from the centroid of each microglial cell, represented in FOV4, and plotted for the entire grey matter hippocampus arranged along the binned MSC bin (180 bins) from low, middle to high MSC. (G) The pixel clusters of the deep white matter of the hippocampus overlay across the acquire FOVs (top) with microglia depicted spatially and colored by their MSC. Pixel cluster frequencies around microglia along the MSC for the deep white matter in the hippocampus (bottom). (H) Correlation (positive and negative) of pixel clusters progression with MSC progression for each healthy brain region in the grey (left) and deep white (right) matter.

To appreciate the distribution and cellular niches of the microglial phenotypic states, we next spatially mapped them back onto tissue. Brain textures surrounding microglia are composed of astrocytes, neurons, synapses, and vasculature and form subregions within larger brain regions. Due to the highly irregular shapes of these features, lack of robust membrane markers and seemingly covering large unbounded spaces, they are difficult to segment using standard nuclear expansion segmentation approaches used in immune tissue. Thus, to overcome these limitations and uncover common signatures of microenvironments across the brain, we used unbiased, automated pixel clustering^60^ across images from brain regions (including grey and white matter) and identified 20 pixel clusters corresponding to unique textures in the tissue using neuropathology lineage features (Figure 5A). These pixel clusters included regions dominated by proteins that were expressed widely in the extracellular space, with intermediate to high excitatory synaptic density (cluster 1: CD56^high^ VGLUT1^pos^, cluster 2: CD56^pos^ VGLUT1^high^), APOE and CD56 expression (cluster 3: APOE^pos^CD56^pos^, cluster 11: APOE^high^), dense white matter areas (cluster 9: MCNPase^high^), mixed white and grey matter areas (cluster 5: MCNPaseposCD56^pos^), and areas with dense mitochondrial protein and free iron (cluster 7: VDAC1^hi^Fe^hi^). Pixel clustering also identified nuclei (cluster 4) and cells, including neuronal soma (cluster 10: RNA^high^HH3^low^), microglia (cluster 8), intravascular immune cells (cluster 12), astrocyte cell bodies (cluster 19), as well as vasculature (cluster 13) and astrocyte endfeet (cluster 6). Neuronal structures like dendrites (cluster 20), white matter axons (cluster 15) and grey matter axons (cluster 16) were also clearly identifiable, despite having elongated and irregular shapes. Tau tangles (cluster 18) were found in the hippocampus as well as intracellular Ab_1–42_, a common occurrence in the aged brain.

The proportion of each pixel cluster for each brain region grey and white matter showed that the white matter was indeed homogenously dominated by myelin (MCNPase^high^ cluster 9) while the grey matter areas from each brain region varied in their composition (Figure 5B). All grey matter regions had synaptic textures and the hippocampus, MFG and caudate had higher proportions of VGLUT1^high^ areas (cluster 2). The SN had the highest proportion of VDAC1^high^Fe^high^ areas while the hippocampus had a small proportion and other brain regions did not have any, suggesting that, in this case, the SN was a large reservoir of accumulated free iron. The cerebellum had the largest proportion of neuronal soma (cluster 10), which corresponded to the densely packed granular layer (**Figure S1B**). The hippocampus, MFG and SN had MAP2^pos^ dendrites (cluster 20) and some (< 25%) white matter textures (clusters 5 and 9). All 20 pixel clusters are shown in the hippocampus in a composite image (Figure 5C) with an enlarged area (FOV1) corresponding to the one shown in Figure 1C, as well as enlarged areas depicting a large blood vessel with intravascular immune cells (FOV2) and the white matter axons and myelin of the alveus (FOV3). Individual pixel clusters are visualized within FOV1 (Figure 5D) and within the entire hippocampus (**Figure S5A**). The combined clusters across the SN, MFG, cerebellum, and caudate were also visualized and showed the varying microenvironments of the grey matter (**Figure S5B**).

We next asked whether microglial states along the MSC are preferentially restricted to certain microenvironments across larger brain regions. To do this, we visualized the MSC of all microglia across the entire hippocampus (and other brain regions, **Figure S5B**) colored by their MSC value and saw that low MSC cells appeared to be enriched within specific hippocampal sub-regions like CA1 (Figure 5E). Middle phase MSC cells appeared to be concentrated in CA4 and DG, while high MSC cells were distinctly compartmentalized within areas like the alveus and stratum radiatum which are both myelin and glial rich areas of the hippocampus. This yet unappreciated organized distribution of continuous microglial states within the healthy adult hippocampus suggests that different microglial states might perform different roles in specific local microenvironments related to the composition of that niche.

To fully untangle the niche composition, we sought to computationally and spatially analyze the texture of the microenvironments directly around each microglia. To that end, we quantified the proportion of pixel clusters within a 20 μm radius of the centroid of each microglial cell (Figure 5F). We then aligned the proportion of pixel clustered brain textures and microglia for each of 180 bins along the MSC (Figure 5F). Low MSC cells were surrounded by a high proportion of dense synaptic VGLUT1^high^CD56^pos^ textures (cluster 2) and MAP2^pos^ dendrites (cluster 20), features that were predominantly present in the CA1 and parts of the stratum radiatum, but were also found in CA2, CA3 and CA4 sub-regions. Middle MSC cells tended be surrounded by a higher proportion of the less synaptically dense CD56^high^VGLUT1^pos^ texture (cluster 1), which was predominant in the subiculum and parts of CA4, DG and stratum lacunosum moleculare. As microglia transitioned from middle to high MSC, there was a distinct enrichment of white matter (cluster 9: MCNPase^high^ myelin and cluster 5: MCNPase^high^CD56^pos^) surrounding them and a loss of dense synaptic textures. The cerebellum also showed a distinct enrichment of white matter, white matter axons as well as vasculature surrounding high MSC cells (**Figure S5B**). If low MSC microglia are in a hypoactive, possibly dystrophic state, their association with dense synapses in the CA1 in this healthy aged hippocampus implies that they may not be performing their normal homeostatic roles there. This is in line with the CA1 area being highly susceptible to degeneration in aging^61^ and the gradual increase of senescent microglia with age^57^.

The MFG, a less compartmentalized isocortical brain region, had a higher density of low MSC cells in general and middle phase MSC cells were modestly enriched with surrounding white matter texture. The SN, like the hippocampus, had a higher overall density of high MSC cells and in the SN these had more astrocyte-rich microenvironments around them. The caudate showed the least compartmentalization of microglial states which may be a result of the homogenous nature of the acquired FOVs in this area. Within the deep white matter tracts in the hippocampus that were acquired separately from the large grey matter area, microglia were also more homogenously surrounded by the same microenvironments which were, unsurprisingly, dominated by myelin (Figure 5G) and similar to other deep white matter brain regions (**Figure S5C**). However, high MSC cells in the white matter had a higher proportion of vasculature in their direct vicinity, suggesting that vessel-proximate microglia were more highly activated than microglia further away from vasculature, possibly due to cytokines and other factors that penetrate the blood brain barrier and activate microglia. We calculated the correlation (both positive and negative) of pixel cluster frequency to progression of MSC and saw that the more varied grey matter brain regions had a higher correlation of MSC to specific pixel clusters overall than the homogeneous white matter regions (Figure 5H). Following this pattern, the hippocampus had the highest correlation of MSC progression with pixel cluster frequencies and was the most organized and compartmentalized brain region we analyzed. Pixel cluster changes that correlated with MSC progression supported what we saw previously, namely VGLUT^high/pos^, MAP2^pos^ and MCNPase^high^ rich areas which represent high synaptic, neuronal and myelinated axon density respectively, correlated with changing microglial states between them.

This data driven pixel clustering analysis of local brain microenvironments provides a new model of brain regional cellular composition and localization based on intracellular and extracellular protein expression^60^, aspects that are not captured by traditional cellular segmentation. We uncovered a compartmentalization of microglial cell states within specific microenvironments where the more structured a brain area is, like in the allocortical hippocampus, the more enrichment of polarized microglial cell states are found within them. Microglia had higher MSC in white matter areas and were more likely hypoactive or dystrophic in grey matter areas with high synaptic and neuronal density. Vasculature, regardless of location, usually implied surrounding microglia were more active than those more distant from vasculature. Notably, the CA1 subregion of the hippocampus had the most hypoactive, low MSC cells across the entire hippocampus, a finding that may be relevant for the high susceptibility of this subregion to degeneration in AD and several other diseases of brain.

### Alzheimer’s disease skews microglial states locally around plaques and globally towards low MSC throughout the hippocampus.

**Figure 6.**
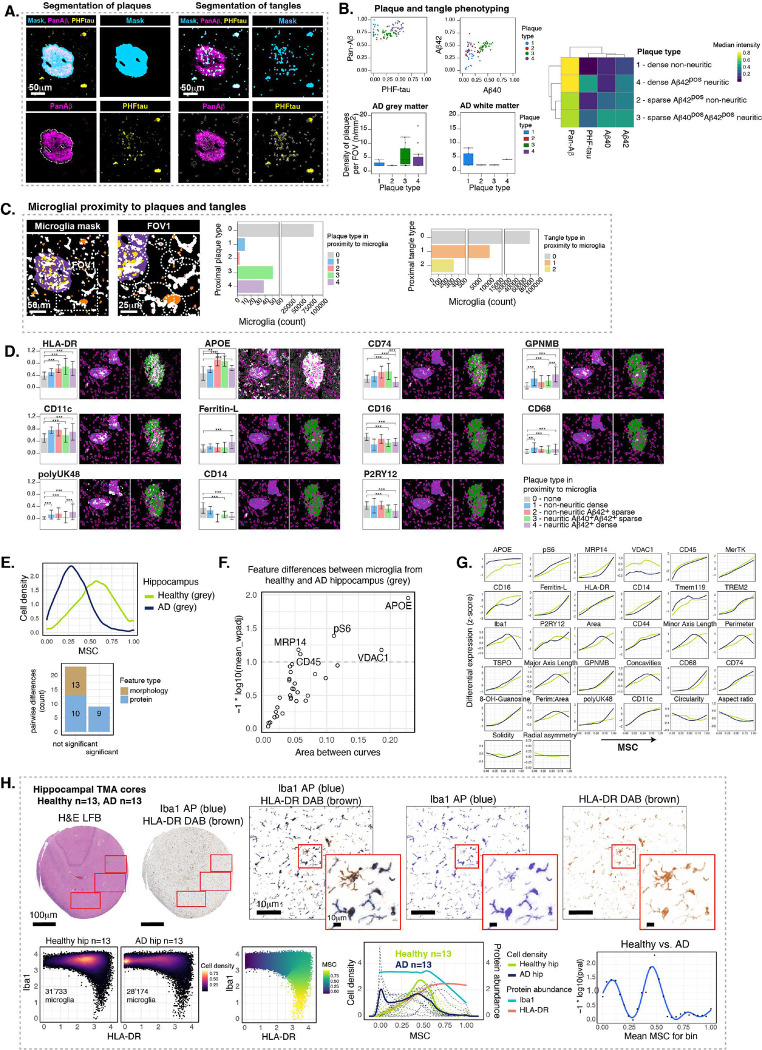
Spatial analysis of microglia in Alzheimer’s disease alters their cellular state according to proximity to amyloid plaques. (A) Segmentation of amyloid plaques (left) and tau tangles (right) from one human AD hippocampus by eZsegmenter through masking (blue) Pan-Aβ (magenta) and PHF-tau (yellow) expression. Human donor details: 82-year-old male with AD dementia, Braak score of V, MMSE score of 19, *APOE* ε3/ε3, and a post-mortem interval of 2.95 hours. (B) Phenotyping of plaque types with FlowSOM clustering (left, top) results in four plaque types with varying levels of Pan-Aβ, Aβ_42_ and Aβ_40_. Density of each plaque type quantified as number of plaques per mm^2^ in the grey and deep white matter of the human AD hippocampus (left, bottom). Relative levels of each plaque phenotyping protein for each plaque type (right). (C) Spatial enrichment analysis of plaques and tangles proximal to single microglia (left) through masking each feature and quantifying the presence of a plaque or tangle within a specified radius (r = major axis length of each cell + 10 pixel buffer) around each microglial mask. Total number of microglia with a plaque or tangle of each type within their direct vicinity (right). (D) Quantification of differentially expressed proteins in disease associated microglia (DAMs) as defined by their proximity to each plaque type, with representative plaque types 3 and 4 shown with microglia overlaid as dashed magenta lines. (E) Microglial cellular density along the MSC between the healthy (green line) and AD (navy blue line) human hippocampus (top) with the total number of statistically significant and not significant pairwise differences within protein and morphology features. (F) Statistically significant feature differences (only proteins) between the healthy and AD human hippocampus as volcano plot, with their PCTBP scores and area between the curves. (G) All feature comparisons (protein and morphology) shown along the MSC for the healthy and AD human hippocampi. (H) Validation of MIBI findings through an independent cohort of 13 healthy and 13 AD hippocampi through low-plex immunohistochemistry: (top) staining the tissue microarray cores of the CA1 region of the hippocampus with Iba1 (blue) and HLA-DR (brown) as a dual stain and *in silico* separation of each protein signal; (bottom) segmentation of microglia through masking both Iba1 and HLA-DR signals together in eZsegmenter and quantification of signal intensity at a single-cell level in each FOV acquired in healthy and AD hippocampus groups. A proxy MSC was calculated with Iba1 and HLA-DR expression from extracted cells (bottom, middle) and the average microglial cellular density plotted for the healthy (green line) and AD (navy blue line) hippocampus (with individual donors in dashed lines) and Iba1 (light blue) and HLA-DR (peach) expression along the MSC. Statistical significance (p-value) was calculated along binned MSCs (bottom, right) with PCTBP scores > 1 interpreted as statistically significant.

Having observed that microglia are better analyzed through a continuum of states, varying between brain regions and within local microenvironments, we sought to assess microglial changes along the MSC within a neurological disease context. We imaged sub-regions of the hippocampus (CA1, CA4, DG) from an AD donor (82-year-old male with AD dementia, Braak score of V, MMSE score of 19, *APOE* ε3/ε3, and a post-mortem interval of 2.95 hours) which included part of the subiculum, CA1, CA4, and DG subfields (**Figure S6A**). Previous studies of microglia in mouse models of AD showed that microglia proximal to Aβ plaques downregulate homeostatic markers like P2RY12 and upregulate activation markers and have been called disease associated microglia (DAMs)^11^. To identify similar niches in human AD, we carried out proximity analysis around plaques that we stained for with pan-Aβ, Aβ_1–40_, Aβ_1–42_ and tau tangles with PHF-tau. We segmented out the plaques and tangles from our images using masks created by pan-Aβ and PHF-tau expression, respectively (Figure 6A) and identified different types of plaques through FlowSOM (Figure 6B, **S6C**). We categorized two major types as dense or sparse plaques by their pan-Aβ intensity. These can be further divided into plaque subtypes with or without PHF-tau^+^ starburst neuritic processes: dense non-neuritic plaques (type 1), sparse non-neuritic plaques with Aβ_1–42_ (type 2), sparse neuritic plaques with Aβ_1–40_ and Aβ_1–42_ (type 3), and dense neuritic plaques with Aβ_1–42_ (type 4). Plaque density was much higher in the grey matter than in the white mater, and plaque types 3 and 4 were most abundant (Figure 6B, 6D). We next made a distinction between PHF-tau^pos^ processes that were present outside of plaques and those that were present within plaques by gating on pan-Aβ^pos^ and PHF-tau^neg^ features (**Figure S6B, S6C**). PHF-tau^+^ tangles were present in both the healthy and AD hippocampus although there was a higher density in the AD case in both the grey and white matter.

In order to determine the phenotype of microglia directly around plaques and tangles, we selected those cells which had a plaque or a tangle nearby (within a specific radius, *see* methods) and quantified the number of cells proximal to each plaque and tangle type (Figure 6C). Since microglia are much more abundant than plaques and tangles, most microglia were not proximal to plaques or tangles (grey bar). Of the microglia that were proximal to plaques, most were nearby neuritic plaques (types 3 and 4). Relatively few microglia were proximal to plaques generally (less than 100 cells in total) compared to tangles (close to 10,000 cells), likely due to the relative abundance of tangles over plaques in the hippocampus of this AD case. We looked to whether microglial phenotype was influenced by proximity to each pathologic feature. We observed that many proteins were changed in comparison to microglia distant from plaques and tangles, including upregulation of HLA-DR, APOE, CD74, CD11c, Ferritin-L, GPNMB, poyUK48 and CD68, and downregulation of CD16, CD14 and P2RY12, (Figure 6D, **S6D**), suggesting a DAM phenotype and therefore supporting observations from previous studies in mice^10,11^. Microglia proximal to dense plaques were shifted towards high MSC states, and therefore more activated, while microglia proximal to sparse plaques were predominantly in middle phase MSC (**Figure S6E**). Microglia that were proximal to tangles were not significantly different in their protein phenotype to microglia that were distant from tangles (**Figure S6F**). Pixel clustering of the AD hippocampus identified, in addition to textures we saw in other healthy brain regions, two types of plaques, namely Aβ_1–40_^pos^ APOE^high^ and Aβ_1–42_^pos^ plaques (**Figure S6E**). High MSC microglia had an enrichment of plaques and vasculature in their direct microenvironment in the grey matter (**Figure S6H**) and an enrichment of vasculature in the white matter (**Figure S6I**), as in healthy brain tissue.

Although we found DAMs around plaques in AD hippocampus, these were comparatively few cells (~100) compared to the total number of cells imaged in the AD hippocampus (~20,000). We asked whether microglia from the AD hippocampus exhibit global changes compared to microglia from the healthy hippocampus. We found that AD hippocampal microglia were globally skewed towards low MSC states while healthy hippocampal microglia were skewed towards middle-high MSC states in the grey matter areas (Figure 6E, **top**), suggesting that microglia from AD hippocampus grey matter were generally less activated and presumably senescent/dysfunctional compared to healthy hippocampal grey matter microglia in this single case. As in other comparisons between healthy brain regions, the statistically significant feature differences were found within the protein rather than morphologic features (Figure 6E, **bottom**). Once again, APOE and VDAC1 were highly statistically significant where AD hippocampal microglia had higher APOE expression and lower VDAC1 expression than healthy hippocampal microglia (Figure 6F, 6G). AD hippocampal microglia had higher levels of p-S6 and lower levels of CD45 than healthy hippocampal microglia. Unique to the AD versus healthy comparison, the trajectory of marker progression changed dramatically at high MSC for several features (Figure 6G). Iba1 decreased suddenly in high MSC in AD microglia, while polyUK48 increased, compared to healthy microglia. Morphological features also changed in high MSC where healthy microglia were the largest and had the highest number of projections in the control hippocampus, but AD microglia were smaller and had fewer projections. Overall, there was a lower density of presumably healthy functional and active microglia in AD, and their state of healthy activation was perturbed. The same was not true for AD and healthy hippocampal white matter microglia (**Figure S6J**), which supports the pathological changes in AD being found predominantly in the grey matter. Indeed, microglia in the grey matter AD hippocampus were skewed towards low MSC compared to microglia in the white matter AD hippocampus (**Figure S6K**).

Since this initial screen was based on a single AD case, we sought to validate our findings of MSC changes in a larger cohort of healthy and AD hippocampal samples. While the multiplexing capacity of MIBI allowed us to uncover the coordinated MSC across multiple proteins and morphologies, we now needed to run many samples with only a few quantified proteins. To do this, we used two-color immunohistochemistry and chose two markers as proxies for the MSC: HLA-DR, which increased from low to high MSC, and Iba1, which decreased from low to high MSC. Thus HLA-DR^low^Iba1^high^ cells would represent low MSC cells and HLA-DR^high^Iba1^low^ cells would represent high MSC cells. We stained a cohort of 13 healthy and 13 AD dementia age-matched hippocampal cores (Figure 6H, *top left*) with Iba1 and HLA-DR on blue and brown chromogens, respectively, and extracted FOVs from CA1 of each core. We then separated the brown and blue colors *in silico* (Figure 6H, *top right*) and segmented out the microglia using the same strategy as for our MIBI data, i.e., creating a composite microglial mask using Iba1 and HLA-DR expression. We extracted approximately 30,000 cells from each of the healthy and AD groups (Figure 6H, *bottom left*). Based on a simple biaxial plot, healthy hippocampal microglia expressed higher levels of HLA-DR than AD hippocampal microglia. We then computed a proxy MSC using Iba1 and HLA-DR which, like our highly multiplexed MIBI MSC, also progressed from high Iba1 and low HLA-DR to low Iba1 and high HLA-DR expression (Figure 6H, *bottom middle*). Indeed, the AD hippocampal microglia were skewed towards low MSC compared to healthy hippocampal microglia which were enriched in the middle MSC, presumably reflecting a healthy active state compared to a potentially senescent inactive state in AD. We calculated progressing p-values along MSC bins (Figure 6H, *bottom right*) and found that differences between healthy and AD MSCs were statistically significant in the low and middle MSC, and not in high MSC, suggesting that AD microglia were indeed globally skewed towards having more inactive HLA-DR^low^ microglia and fewer healthy active HLA-DR^+/high^ middle MSC microglia. However, more activated, high MSC microglia were still present in both healthy and AD hippocampi, which may represent DAMs around plaques in AD and/or microglia in myelin rich niches without pathology.

Finally, we assessed correlation between MSC and MMSE (Mini Mental State Exam), which is a cognitive assessment test taken by all the donors of our cohort towards the end of life, where a score of 24 or less suggests a likely diagnosis of dementia. Rather than grouping samples into two groups, MMSE allowed for a continuous variable association with MSC and correlated positively (R = 0.31, p < 2.2 e-16) where low MSC, corresponding to impaired microglial states, associated with low MMSE score, and higher MSC correlated with higher MMSE score (**Figure S6L**). Overall, our highly multiplexed spatial proteomic MIBI data and chromogenic validation show that microglia from the AD hippocampus, specifically the CA1 sub-region, are globally skewed towards a less expressive, less active, potentially senescent state microglia. These deficient microglia are likely not performing their crucial roles in brain homeostasis and may be contributing to synaptic loss and cognitive decline in this highly susceptible and vulnerable brain area. Microglia from the healthy hippocampus CA1 more actively expressed many proteins and are likely performing compensatory functions that help maintain brain health and cognition in old age. Finally, our data support that differential expression of HLA-DR and Iba1 by microglia can be used to understand cellular state in health and disease, thus providing easy-to-use tools for tracking microglial states in future studies using any human brain tissue.

## Discussion

We employed spatial proteomics and endogenous metal quantification by MIBI to deeply phenotype human microglia across five healthy brain regions and AD hippocampi (Figure 1). We segmented individual microglia (Figure 2–3) and implemented a pseudotime trajectory analysis to reveal a continuum of phenotypic states we termed the Microglial State Continuum (MSC), characterized by a high dynamic range of protein expression (Figure 4). Low MSC microglia had low expression levels for many proteins associated with myeloid cell activation, were smaller and had dystrophic morphology while middle to late microglia had higher to very high expression levels for many proteins and were larger and thicker, resembling healthy activated to highly activated states. Besides density of microglial contribution, inter-brain regional variation of MSC distribution was also seen, where the healthy MFG and caudate were skewed towards low MSC, the cerebellum was enriched in middle MSC and the hippocampus and substantia nigra skewed towards high MSC (Figure 4). Furthermore, we demonstrated that this multi-cellular brain environment’s relationship to the MSC position was not just driven by macro-anatomical features, but also micron-scale, subregional niches using pixel clustering and neighborhood analysis (Figure 5). For instance, we showed enrichment of high MSC microglia within niches of high myelin and axon density as well as close to vasculature, and of low MSC microglia in niches that had high synaptic and soma density, particularly in the hippocampus. We also localized DAMs in direct contact with amyloid plaques in AD hippocampus, which were enriched in middle to high MSC, away from the most activated states we observed (Figure 6). These changes in MSC-based localization as a function of both anatomical space and proteopathy suggest a link towards cell (dys)function, and reinforce the idea of uniquely diverse and plastic human microglia with specifically tuned roles in brain homeostasis^62^.

Notably, in the AD hippocampus, microglia spatially uninvolved with plaques and tangles vastly outnumbered DAMs (i.e., ≫ 99:1) and exhibited a low, presumably inactive, MSC phenotype, suggesting a dystrophic and perhaps senescent and impaired functional state. This overall low-shifted MSC persisted when we validated our MIBI findings across an independent cohort of 13 AD and 13 control hippocampi by targeted low-plex IHC, quantifying the co-expression of Iba1 and HLA-DR (Figure 6). Like other aspects of neurodegeneration, it will be important to understand if shifts in these microglial states are causal to the disease process or a response to the degeneration process. If causal, the expectation is that similar shifts may be present of other degenerative processes with associated proteopathy, like Parkinson’s disease. Moreover, the anatomical influence on MSC suggests that caution should be taken when assessing glial cells from one brain region and making conclusion on their influence in another.

While most proteomic imaging and single cell analysis techniques tend to binarize molecules of interest into positive and negative cells and pixels, this approach would miss important, but less obvious, changes of microglial protein expression. Our application of MIBI allowed the quantification of proteins across a dynamic range on a cell/pixel basis allowing us to capture a progression of microglial states based on gradual expression changes along a spectrum which corresponds both to anatomical biases as well as healthy vs. impaired cognitive states. Microglial molecular phenotypes, whether proteomic or transcriptional, are no longer viewed as discrete populations but rather as overlapping and progressively changing in response to an onslaught of stimuli^63,64^. Furthermore, spatial information is critical to understand the diversity of microglia present in the brain. Mouse studies focus on specific DAM states around amyloid plaques which are often not found in human studies, largely due to the lack of single-cell and spatial resolution. Our data demonstrated that human microglia are indeed able to transition to the DAM state, which is considered protective in AD, however, it also shows this represents only a small fraction of microglia that are in direct contact with plaques undergo this transition. Instead, we see that most microglia in the human AD hippocampus are not in contact with plaques or tangles but appear to instead be globally shifted to a dystrophic or impaired state. Other studies of human aged and AD brain regions have shown microglia to have an accelerated aging profile in AD (HAMs, Human AD Microglia)^14^ rather than a DAM profile, although the DAM signal was likely lost in the bulk analysis, so this likely reflects the global microglial switch to an impaired response. Altogether, this study framework provides a single cell lens through which to view human microglial diversity *in situ* that seems not to be limited to those cells perturbed by disease pathology interaction, but also global, anatomically specific shifts that could be used to understand dysfunction in disease and are a potential point of therapeutic intervention.

## Methods

### Human brain tissue

Human FFPE brain samples that was imaged by MIBI were acquired from the Arizona Study of Aging and Neurodegenerative Disorders and Brain and Body Donation Program at Banner Sun Health Research Institute (brainandbodydonationprogram.org)^68^. Brain regions including the hippocampus, cerebellum, substantia nigra, caudate nucleus and middle frontal gyrus were from a healthy donor who was a 90-year-old male with no dementia, an MMSE score of 27, Braak stage I, *APOE* genotype ε3/ε3, and a post-mortem interval of 3.4 hours. The AD dementia case imaged by MIBI was the hippocampus from an 82-year-old male with MMSE score of 19, Braak stage V, *APOE* genotype ε3/ε3, and a post-mortem interval of 2.95 hours. The tissue microarray used to validate MIBI findings comprised 26 hippocampal human FFPE brain cores selected from patients of The 90+ Study cohort (https://doi.org/10.1002/alz.12981) whose pathologic evaluation was performed at Stanford Pathology Department. Donors were age-matched and selected based on their clinical diagnosis and neuropathological scores evaluated by NIA-AA guidelines^65^. The donor details are listed in **Extended Data Table S2**.

### Antibody preparation

Antibodies were first screened by IHC on FFPE brain sections to select for the best clones. These were then conjugated to isotopic metal reporters as described previously^40,44^. Following conjugation antibodies were diluted in Candor PBS Antibody Stabilization solution (Candor Bioscience). Antibodies were either stored at 4°C or lyophilized in 100 mM D-(+)-Trehalose dehydrate (Sigma-Aldrich) with ultrapure distilled H2O for storage at −20°C. Before staining, lyophilized antibodies were reconstituted in a buffer of Tris (Thermo Fisher Scientific), sodium azide (Sigma-Aldrich), ultrapure water (Thermo Fisher Scientific) and antibody stabilizer (Candor Bioscience) to a concentration of 0.05 mg ml^−1^. The antibodies, metal reporters and staining concentrations are listed in **Extended Data Table S1**. For detailed metal-antibody protocol MIBItag see dx.doi.org/10.17504/protocols.io.bhyej7te.

### Tissue staining

FFPE brain tissues were sectioned (5μm section thickness) from tissue blocks on gold and tantalum-sputtered microscope slides. Slides were baked at 70°C overnight, followed by deparaffinization and rehydration with washes in xylene (3×), 100% ethanol (2×), 95% ethanol (2×), 80% ethanol (1×), 70% ethanol (1×) and ddH2O with a Leica ST4020 Linear Stainer (Leica Biosystems). Slides next underwent antigen retrieval by submerging sides in 3-in-1 Target Retrieval Solution (pH 9, DAKO Agilent) and incubating at 97°C for 40 min in a Lab Vision PT Module (Thermo Fisher Scientific). After cooling to room temperature for 1 h, slides were washed in wash buffer (1× PBS IHC Washer Buffer with Tween 20 (Cell Marque) with 0.1% (w/v) bovine serum albumin (Thermo Fisher)). Next, all slides underwent two rounds of blocking, the first to block endogenous biotin and avidin with an Avidin/Biotin Blocking Kit (BioLegend). Slides were then washed with wash buffer and blocked for 1 h at room temperature with 1× TBS IHC Wash Buffer with Tween 20 with 3% (v/v) normal donkey serum (Sigma-Aldrich), 0.1% (v/v) cold fish skin gelatin (Sigma-Aldrich), 0.1% (v/v) Triton X-100, and 0.05% (v/v) Sodium Azide. The first round of staining was done with free indium 115^3+^ (8 mM diluted in PBS, Fluidigm) in staining buffer (1x TBS IHC Wash Buffer with Tween 20 with 3% (v/v) normal donkey serum) and incubated overnight at 4°C in a humidity chamber. The following day, slides were washed twice for 5 min on a shaker in wash buffer. The second round of staining was done using the cocktail of metal conjugated antibodies prepared in staining buffer at their respective concentrations and filtered through a 0.1 μm centrifugal filter (Millipore) prior to incubation with tissue overnight at 4°C in a humidity chamber. Following the overnight incubation with the antibody cocktail, slides were washed twice for 5 min in wash buffer. On the third day, anti-biotin 152 Eu was prepared as described and incubated with the tissues for 1 h at 4°C in a humidity chamber. Following staining, slides were washed twice for 5 min in wash buffer and fixed in a solution of 2% glutaraldehyde (Electron Microscopy Sciences) solution in low-barium PBS for 5 min. Slides were then washed in PBS (1×), 0.1 M Tris at pH 8.5 (3×) and ddH2O (2×) and then dehydrated by washing in 70% ethanol (1×), 80% ethanol (1×), 95% ethanol (2×) and 100% ethanol (2×). Slides were dried under vacuum overnight prior to imaging. For the detailed staining protocol see dx.doi.org/10.17504/protocols.io.dm6gprk2dvzp/v5.

### Image acquisition on MIBI

Prior to imaging, slides were sputter coated with 10 nm of gold over the entire stained tissue section on each slide in order to ensure no charging effects of the tissue which impact on field uniformity during imaging. Imaging was performed using a MIBI instrument with a Hyperion ion source by sequential rastering: pre-rastering at half the ion dose to remove the gold coating on FOVs of interest, followed by final rastering and image collection at full ion dose. Xe^+^ primary ions were used to sequentially sputter pixels for a given FOV. The following imaging parameters were used: aperture setting, 300 μm; acquisition setting of 100 kHz; FOV size, 700 μm × 700 μm at 1,024 ×1,024 pixels per FOV; sample bias, 20 V (pre-raster, ions not funneled into the TOF chamber to preserve the detector) and 50 V (final raster, ions funneled into the TOF chamber); dwell time, 0.5 ms (pre-raster) and 1 ms (final raster for image acquisition); median gun current on tissue, 13.6 nA; an ion dose of ~15 nAmp.ms.nm^−2^ (pre-raster) and ~30 nAmp.ms.nm^−2^ (final raster). Acquisition time was 8.7 minutes per FOV (pre-raster) and 17.33 minutes per FOV (final raster). FOVs were tiled across each brain region with an overlap of 50 μm in the *x* and 20 μm in the *y* direction. A total of 420 FOVs were acquired across all brain regions, including grey and deep white matter.

### Low-level image processing

Multiplexed image sets were extracted, slide background-subtracted, denoised and aggregate filtered as previously described^40,44^, using Matlab scripts and in-house GUIs for MIBI image processing. Additionally, non-specific binding to charged neurons (due to over fixation of tissue in formalin prior to embedding in paraffin blocks) was subtracted by masking neurons using the indium 115^3+^ signal and removing signal from channels that contained non-specific binding. For visualization purposes, individual FOVs were stitched together to reconstruct large areas of each brain region using the Fiji/ImageJ image processing environment and existing plugins.

### Microglial and pathology segmentation

To segment microglia, amyloid plaques and PHF tau tangles we used EZSegmenter, a MATLAB regionprops thresholding-based segmentation GUI developed in-house and available as a part of the MIBI image processing toolkit here: https://github.com/angelolab/MAUI, as previously described^44^. Briefly, multiplexed TIF images from multiple FOVs are loaded into the GUI. Microglial masks were created using the combined Iba1 and CD45 protein expression, amyloid plaque masks were created using pan-Aβ protein expression and PHF tau tangle masks were created using PHF tau protein expression. Parameters for masking are adjusted for each mask separately (e.g. Gaussian Blur, minimum and maximum object pixel size) and fixed across all FOVs. Masks are then used to extract pixel-level signal intensities across each channel and then cell or object size normalized before import into an output cell table csv file. Single cell or object data was then imported into R for arcsin h transformation and normalization.

### Microglial single-cell computational analysis

#### Clustering

Protein and morphology features of segmented microglial cells were mean-centered and scaled, and unsupervised clustering was performed on protein-only features using FlowSOM^58^ v2.2.0 in R version 4.1.3.

#### Computation of the microglial state continuum

The microglial state continuum (MSC) was generated using the SCORPIUS^59^ algorithm with a lowess smoother and 1000 iterations. Trajectory inference was performed using either protein-only, or morphology-only features. Microglia from the HIP, Caudate, Cerebellum, SN, and MFG brain regions were included (grey and white matter), and features were mean-centered and scaled prior to trajectory inference. Trajectory inference was also performed on microglia from individual brain regions and a spearman rank correlation was used to compare the pseudotime estimate of each individual region, to the all-region MSC, which had strong agreement with each other.

#### Estimating microglia state in the AD hippocampus using machine learning

To estimate the MSC of microglia in the AD hippocampus, we used kernel support vector machine (KSVM) with epsilon regression and a radial basis function Gaussian kernel. KSVM was done using the ksvm() function in the R package kernlab^66^ v0.9.30 with parameters type = ‘epssvr’ and kernel = ‘rbfdot’. To validate predictive accuracy of the model, we used 5-fold cross-validation. Microglia data from all regions except AD hippocampus was randomly split into 5 folds and SCORPIUS was computed on the training set for each fold. The KSVM model was trained with protein features and pseudotime as the response variable, and then prediction was performed on the hold-out set. We compared the predicted pseudotime to the pseudotime computed separately on the hold-out set. Model performance was assessed using correlations and residuals. Predicted and true pseudotime estimates were significantly correlated with values between 0.99 and 1.0, residuals were randomly distributed along the fitted line, and absolute mean and standard deviation of residuals across all folds was less than 1.8% and 0.02%, respectively.

#### Pairwise Comparison of Trajectories by Binned Permutations (PCTBP)

To compare protein and morphology expression between microglia from different brain regions along the MSC in a pairwise manner, we developed a non-parametric statistical method using binned permutations along the trajectory with 3 major steps.

1. Calculate the optimal bin width using Freedman-Diaconis rule^67^.
2. Perform permutation analysis: Randomly scramble, without replacement, the pair of labels to compare. Within each bin, calculate the mean difference between the scrambled labels. Compare the absolute permutation mean difference from the scrambled labels to the absolute true difference (test statistic) between the true labels in the same bin. Repeat this step 1000x times. The permutation score is the proportion of samples that have a test statistic at least 1.5x greater than the permutation mean difference. A minimum 1.5x difference is required to improve robustness of the method by incorporating a fold change threshold.
3. The permutation score of each bin along the trajectory is corrected for multiple comparisons and smoothed using a locally estimated scatterplot smoothing and the final PCTBP score is the average smooth permutation score along the trajectory.

The bin-based permutation approach to generating the PCTBP score allows for flexible analysis that can capture both local and global differences along the trajectory. The PCTBP score is generated using permutation scores along the entire binned continuum, unless otherwise noted. When comparing low, middle, and high parts of the continuum, smoothing is performed prior to splitting the low, middle, and high PCTBP scores.

### Pixel clustering and spatial enrichment analysis

Pixel clusters were identified using the Pixie pipeline^60^. Briefly, single-pixel expression profiles were extracted from pre-processed MIBI images from all brain regions combined. A Gaussian blur was applied using a standard deviation of 2 for the Gaussian kernel. Pixels were normalized by their total expression, such that the total expression of each pixel was equal to 1. A 99.9% normalization was applied for each marker. Pixels were clustered into 100 clusters using a self-organizing map (SOM) based on the expression of 17 markers: HH3, CD56, Indium, APOE, CD45, CD105, NEFL, NEFH, VDAC1, MCNPase, VGLUT1, Iba1, MAP2, S100β, GFAP, pan-Aβ and PHFtau. The average expression of each of the 100 SOM clusters was found and the z-score for each marker across the 100 SOM clusters was computed. All z-scores were capped at 3, such that the maximum z-score was 3. Using these z-scored expression values, the 100 SOM clusters were hierarchically clustered using Euclidean distance into metaclusters. These metaclusters were manually adjusted and mapped back to the original images such that each pixel was assigned to one pixel cluster. To characterize the microenvironment in direct vicinity of each microglial cell, we defined a radius of 40 pixels (20 μm) from the centroid of each microglial mask and calculated the proportion of pixel clusters within. Microglia were binned into 180 bins along the MSC for the hippocampus, and 80 bins for the other brain regions. Average pixel cluster proportions were calculated for the cells within each bin and plotted in ascending order along the MSC.

### Pathology and DAM analysis

Plaques were clustered using FlowSOM based on pan-Aβ, Aβ_1–40_, Aβ_1–42_ and PHF-tau expression within them. To identify microglia that localized directly around plaques and tangles (i.e. DAMs), we bucketed microglia containing masked plaques or tangles within a set radius from each cell centroid. The radius was determined by taking the major axis length of each cell and adding a 10-pixel buffer to ensure only those cells in relatively close proximity to pathology would be considered DAMs. Additional buffers, including 50 and 100 pixels were tested (data not shown), but lost the ability to separate out the majority of microglia into DAM or not.

### Immunohistochemistry

For IHC screening of panel antibodies, FFPE human hippocampal tissue was sectioned onto standard glass slide at 5 μm thickness. Slides containing tissue were baked at 70 °C overnight. Tissue sections were then processed and stained using the sequenza method with single primary antibody. The IHC protocol mirrors the MIBI protocol, with the addition of blocking endogenous peroxidase activity with 3% (v/v) H_2_O_2_ (Sigma-Aldrich) in ddH2O after epitope retrieval. On the second day of staining, instead of proceeding with the MIBI protocol, tissues were washed twice for 5 min in wash buffer and stained using ImmPRESS universal (Anti-Mouse/Anti-Rabbit horse radish peroxidase) kit (Vector Laboratories). For detailed IHC staining techniques see dx.doi.org/10.17504/protocols.io.bf6ajrae and dx.doi.org/10.17504/protocols.io.bmc6k2ze.

For the double antibody staining of the validation cohort, sections were baked at 70°C overnight followed by deparaffination and antigen retrieval (Dako; S2367). Endogenous horseradish peroxidase (HRP) and alkaline phosphatase (AP) were blocked by applying BLOXALL (Vector Laboratories; SP-6000–100) for 30 minutes followed by blocking buffer solution as for sequenza for 1 h at room temperature. Antibodies Iba1 (clone: EPR16588; cam; ab220815) and HLA-DR (clone: CD3/43; Abcam; ab7856), both diluted to 1 μg/mL in 3% (v/v) normal horse serum, were added together to each section and incubated at 4°C overnight. After washing three times in wash buffer (95% 1xTBS IHC Wash Buffer with Tween 20), secondary antibodies (ImmPRESS Duet Reagent: HRP Anti-Mouse IgG and AP Anti-Rabbit IgG; Vector Laboratories) were added for 10 minutes at room temperature followed by three washes with wash buffer. Antibodies were revealed with Vector Blue AP substrate (Vector Laboratories; SK-5300) for 5 minutes followed by DAB HRP substrate (Vector Laboratories; SK-4105) for 40 seconds. For detailed protocols see dx.doi.org/10.17504/protocols.io.81wgbyoryvpk/v1.

### Software, data and code availability

Software for running the MIBI equipment was developed by SAI (MiniSIMS 2 Data Systems). The code for the analysis can be downloaded at https://github.com/bryjcannon/MIBI_Brain_Analysis. All the information required for cell and object segmentation are available at Ark-Analysis https://github.com/angelolab/ark-analysis. All imaging data and analysis annotations will be made available in a public repository with a fixed DOI # upon peer reviewed publication.
